# Evaluating Adsorbate–Solvent Interactions: Are Dispersion Corrections Necessary?

**DOI:** 10.1021/acs.jpcc.3c02934

**Published:** 2023-05-19

**Authors:** Eleonora Romeo, Francesc Illas, Federico Calle-Vallejo

**Affiliations:** † Departament de Ciència de Materials i Química Física & Institut de Química Teòrica i Computacional (IQTCUB), Universitat de Barcelona, C/Martí i Franquès 1, 08028 Barcelona, Spain; ‡ Nano-Bio Spectroscopy Group and European Theoretical Spectroscopy Facility (ETSF), Department of Polymers and Advanced Materials: Physics, Chemistry and Technology, University of the Basque Country UPV/EHU, Av. Tolosa 72, 20018 San Sebastián, Spain; § IKERBASQUE, Basque Foundation for Science, Plaza de Euskadi 5, 48009 Bilbao, Spain

## Abstract

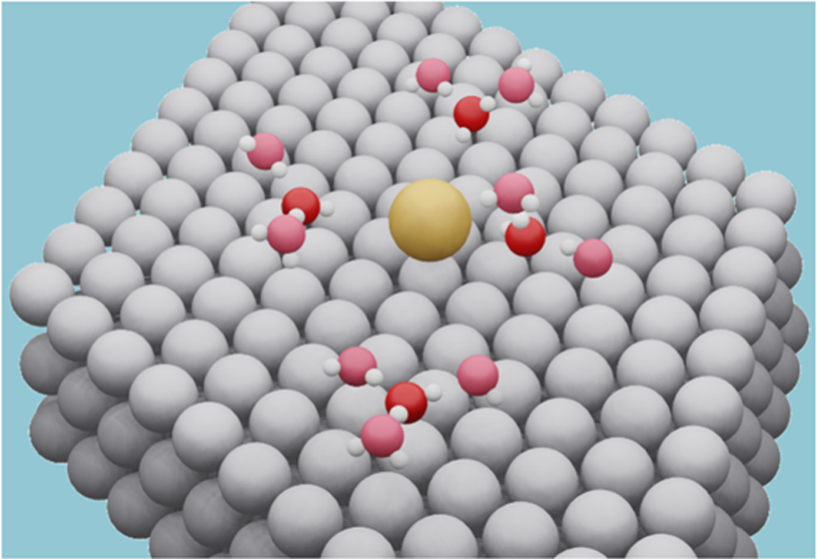

Incorporating solvent–adsorbate interactions is paramount in models of aqueous (electro)catalytic reactions. Although a number of techniques exist, they are either highly demanding in computational terms or inaccurate. Microsolvation offers a trade-off between accuracy and computational expenses. Here, we dissect a method to swiftly outline the first solvation shell of species adsorbed on transition-metal surfaces and assess their corresponding solvation energy. Interestingly, dispersion corrections are generally not needed in the model, but caution is to be exercised when water–water and water–adsorbate interactions are of similar magnitude.

## Introduction

Electrocatalytic reactions are largely influenced by the interactions between adsorbates, substrates, solvents, and electrolytes.^1−3^ Despite its importance, the investigation of solvent–adsorbate and solvent–substrate effects in electrocatalysis is still in its infancy, especially for aqueous solutions and surfaces with defects.^4−16^ To describe solvent–adsorbate interactions, one can resort to implicit solvent, microsolvation, and explicit solvent methods. The way the solvent is described varies from one family of methods to the next and so do the accuracy and the computational expenses. Implicit methods represent the solvent as a homogeneous and constant dielectric continuum around the adsorbate.^17,18^ While this group contains the cheapest computational approaches, its limitations are salient for describing local and directional adsorbate–solvent interactions such as hydrogen bonding.^11,19,20^

In microsolvation methods, explicit water molecules from the first solvation shell(s) of the adsorbate are included, enabling a better local description of hydrogen bonding.^11,21,22^ In some cases, microsolvation can be used in combination with implicit solvation.^18,23^ A critical point in these affordable approaches is the correct positioning and orientation of the explicit solvent molecules, which can be addressed by optimizing several initial configurations.

Water bilayer models are a special type of microsolvation approach for transition metals consisting of an icelike hexagonal water structure above a close-packed surface.^5,6,24^ The idea originated in 1982 from a study that proposed a bilayer model for extended two-dimensional (2D) overlayer adsorption of water on Ru(0001).^25^ In 1994, it was observed that the best fit to the measured data with D_2_O was when the O atoms are almost coplanar, which is inconsistent with the bilayer model where O atoms are buckled by about 1 Å.^26^ A computational study in 2002 suggested that the buckling was a sign of a partially dissociated water layer with intercalated *OH in the hexagons.^27,28^ Although water bilayers are more affordable than a full description of the solvent, a plethora of local minima may be found, and there are difficulties in applying the concept on nanoparticles^21,22^ and surfaces with defects. In fact, various studies have shown a range of structures on different surfaces, such as isolated water clusters, one-dimensional (1D) chains, and 2D overlayers with different sizes and shapes.^4,29−31^

In explicit methods, large portions of the solvent are incorporated in the calculations. An affordable alternative within this family is provided by hybrid quantum mechanics/molecular mechanics (QM/MM) models,^18,32^ where the atoms involved in the reaction coordinate (*i.e.*, surface, adsorbate and its first solvation shells) are treated using quantum mechanics, while the environmental zone (*i.e.*, bulk solvent molecules) is described using molecular mechanics. The latter part of the system is highly dependent on the choice of force field, and numerous options are available. The challenge also remains as to where and how to define the boundary between the QM and MM subsystems.^18,33^

Ab initio molecular dynamics (AIMD) is an explicit method including large numbers of solvent molecules. It gives dynamic information on the interaction between the electrode and solution, as well as the effects of electronic polarization, which can be helpful in modeling electrocatalysts.^12,34,35^ AIMD is computationally demanding, and the data analysis can be arduous. Hence, it is still uncommon in computational electrocatalysis, especially for nanoparticles.^36^

Seeking a compromise between computational cost and accuracy, a microsolvation approach was recently proposed for oxygenate adsorbates with interfacial water molecules.^21,22^ Considering only the water molecules in the first solvation shell, which is usually three for *OH and *OOH, the approach renders results in agreement with those of water bilayers and AIMD for the solvation energies of those two adsorbates. Recently, the method was enabled to evaluate the energy stabilization of any adsorbate at metal surfaces induced by solvent–adsorbate hydrogen bonds, and good agreement was found between calculated and experimental onset potentials of CO_2_ reduction and akin reactions on transition metals.^11^ The method allows to calculate the actual number of water molecules in the first solvation shell of the adsorbate by iteratively comparing water–adsorbate and water–water interactions. Hence, the latter interactions are crucial, as they allow to determine if and how a water molecule takes part of the first solvation shell of the adsorbate.

With all this in mind, we focus in this work on interfacial water–water interactions, hereon referred to as “water self-solvation”, on the (111) and (100) facets of nine transition metals (Co, Ni, Cu, Rh, Pd, Ag, Ir, Pt, Au). Subsequently, we exemplify the use of the microsolvation method. We show that the water self-solvation and adsorbate solvation energies calculated with and without a D3 estimate of dispersion^37^ do not differ considerably, which is counterintuitive, as bulk water modeling usually requires those.^38^ This finding is valuable, as water is generally weakly adsorbed on the basal planes of transition metals, while other adsorbates such as *NO, *NOH, *NHO, *CO, *OH, etc. are strongly chemisorbed. When water is coadsorbed with those species, it is important in (electro)catalysis modeling that the adsorption energies are not overestimated, which might be the case when using dispersion corrections.

## Results and Discussion

**Figure 1:**
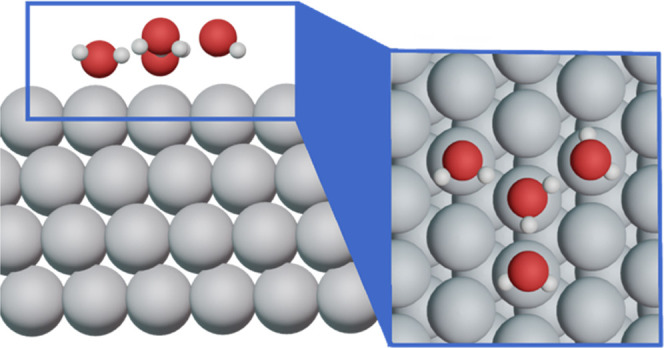
Side (left) and top (right) views of a cluster of four water molecules. The central water molecule is solvated by the three peripheral ones in the first solvation shell. See more configurations in Figure S2.

Full computational details appear in Section S1 of the Supporting Information. Before continuing, we emphasize that all terms in the right-hand side of the equations that follow come from density functional theory (DFT) calculations in which the atoms in the topmost layers and the adsorbates have been fully relaxed (see further details in Section S1). Water self-solvation is assessed using a cluster of four water molecules adsorbed on a metal surface. In the cluster, we distinguish between the central molecule and the three peripheral molecules solvating it (Figure 1). The stability of the central molecule is evaluated both in the solvated environment and in vacuum using eqs 1 and 2, respectively
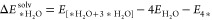
1
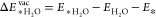
2where *E*_[*H_2_O + 3*H_2_O]_ is the energy of the surface with an adsorbed water molecule solvated by three peripheral water molecules (see Figures 1 and S2), *E*_H_2_O_ is the energy of a single, free water molecule, and E_*H_2_O_ is the energy of its adsorbed state. *E*_4*_ and E_*_ are the energies of the sites where the solvation and adsorption processes occur, respectively. The difference between eqs 1 and 2 is referred to as the total self-solvation energy of water
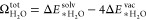
3

In principle, *E*_4*_ and E_*_ in eq 2 are both the total energies of bare slabs and may or may not be of the same size. Because three peripheral water molecules solvate the central one by making three hydrogen bonds in the process, we obtain the self-solvation energy per hydrogen bond by dividing eq 3 by three
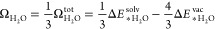
4

In eq 4, Ω_H_2_O_ is the self-solvation energy used as a decision criterion to iteratively determine the number of water molecules solvating a given adsorbate *A (Ω*_A_*).^11^ Specifically, if Ω_H_2_O_ > Ω_A_^1H_2_O^, then *A* is solvated by at least one water molecule, and another water molecule is added to continue the test. This will be illustrated in depth later in the text. Since the mass conservation principle is fulfilled in eq 3, such that *E*_4*_ = 4E_*_, it can be mathematically shown that a simplified way of writing eq 4 is , where *E*_4*H_2_O_ is the energy of four adsorbed water molecules with no hydrogen bonds among them. For completeness, we evaluated the final self-solvation energy in eq 4 by averaging the Ω_H_2_O_ arising from different configurations of *E*_[*H_2_O + 3*H_2_O]_. The average is made from the most stable configurations upon relaxation of the water clusters starting from three ansatzes. Specifically, the central water molecule is initially placed (i) with the two O–H bonds parallel to the surface, (ii) with one of the H atoms pointing away from the surface plane, and (iii) with one of the H atoms pointing toward the surface plane (see Figure S2).

**Figure 2:**
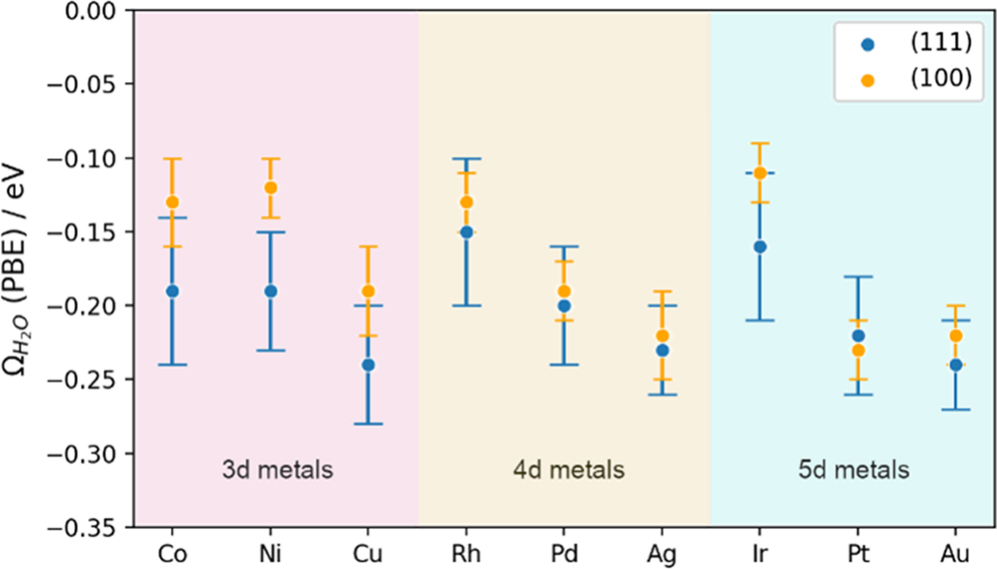
Water self-solvation energies (Ω_H_2_O_) for the (111) and (100) facets of nine metals calculated with PBE. Specific values and associated error bars appear in Tables S2 and S3.

The calculated values of Ω_H_2_O_ are plotted in Figure 2 and reported in Tables S2 and S3 for the (111) and (100) facets of nine transition metals. For a given metal, Ω_H_2_O_ is generally more negative on the (111) facet compared to the (100), except for Pt. We attribute the less negative values of Ω_H_2_O_ on the (100) facet to the stronger adsorption of water molecules, which weakens the strength of hydrogen bonds. The adsorption energies of water are reported in Tables S7 and S8 and Figure S7.

**Figure 3:**
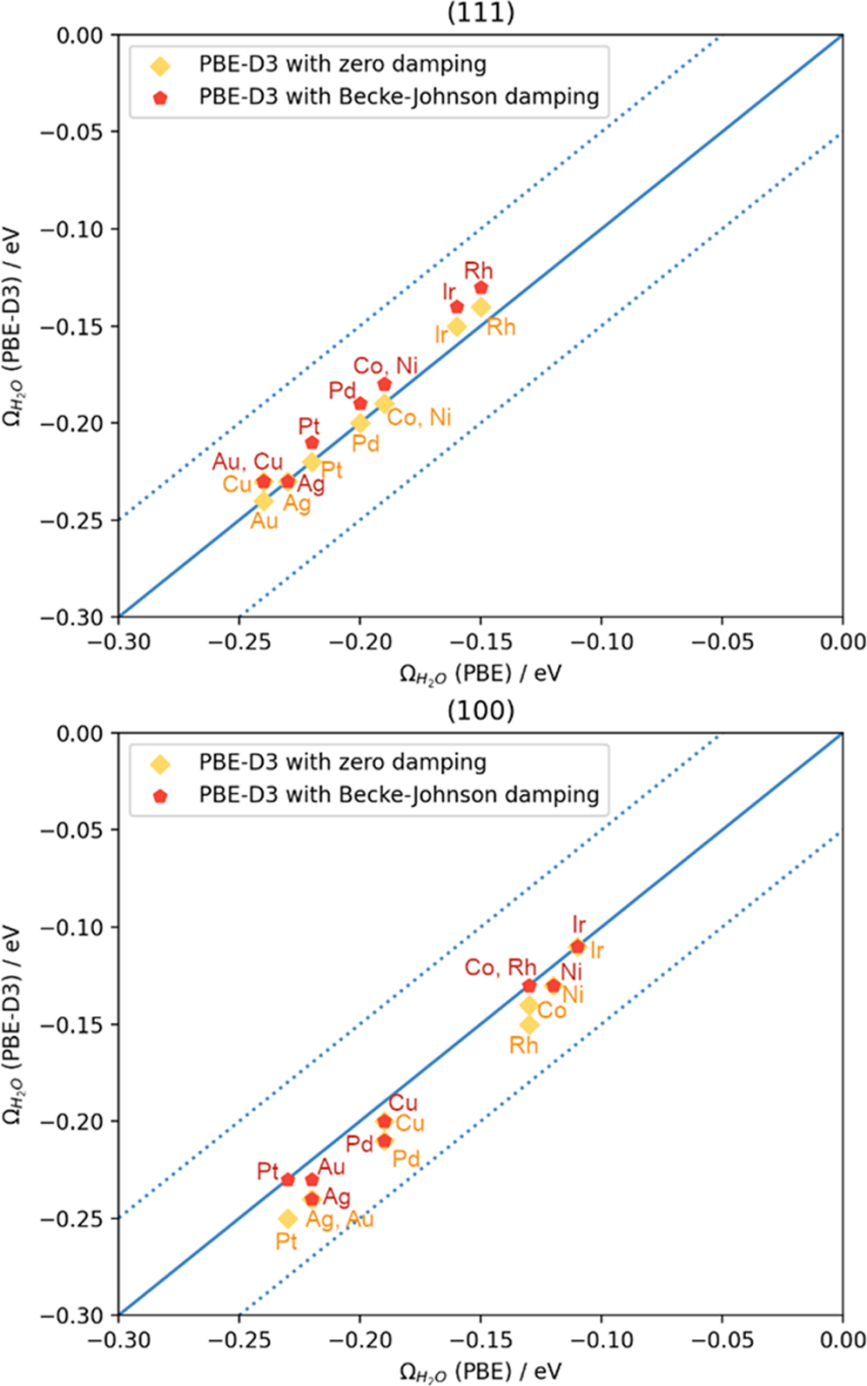
Parity plots of the water self-solvation energy for (top) (111) and (bottom) (100) facets. The plots compare the plain PBE values against those with PBE-D3 dispersion corrections with zero damping (orange) and BJ damping (red). All data fall within the area marked by the dotted lines, which are at ±0.05 eV of the parity line. The data in this figure are reported in Tables S2 and S3.

We compared the results of water self-solvation obtained by means of DFT calculations with the PBE^39^ functional to those obtained with PBE including dispersion through the D3 (hereon referred to as PBE-D3) method by Grimme et al.,^37^ with the zero damping function and with the Becke–Johnson (BJ) damping function.^37,40^ In the adsorption energies of water in Tables S7 and S8 and Figure S8, we note fairly constant negative shifts of −0.22 ± 0.02 eV for the (111) surfaces and −0.20 ± 0.02 eV for the (100) surfaces when dispersion contributions are incorporated. Nonetheless, the water self-solvation energies computed with PBE-D3 are not far from those obtained with plain PBE. In fact, the corresponding deviations shown in Figures 3 and S4 are usually around ±0.02 eV both with zero damping and BJ damping, which is small enough to assert that including dispersion effects is generally not necessary for evaluating the self-solvation energy of water. This is a relevant and nontrivial conclusion, as calculations of bulk liquid water using GGA exchange–correlation functionals habitually require dispersion to avoid over-structuring and wetting problems.^38^

In the following, we explain the procedure to assess the stabilization granted by adsorbate–solvent hydrogen bonds using microsolvation.^11^ As said before, the process involves the consecutive addition of water molecules around the adsorbate in configurations that enable the formation of hydrogen bonds.

The aforementioned process for an adsorbate *A solvated by *n* water molecules can be written as
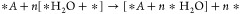
5

We note that the terms in eq 5 are schematized in Figure S5, and the brackets represent solvated surface states. The solvation energy (Ω*_A_*^*n*H_2_O^) is described by
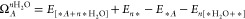
6where *E*_n*_ is the energy of the surface where the process occurs with no adsorbates or water over it. In analogy to eq 3, it has been shown that eq 6 can be calculated as Ω_*A*_^*n*H_2_O^ = Δ*E*_*A*_^*n*H_2_O^ – Δ*E*_*A*_, where Δ*E*_*A*_^*n*H_2_O^ and Δ*E*_*A*_ are the adsorption energies of *A* with and without *n* water molecules in the surroundings.^11^ After each water molecule addition starting with *n* = 1, the gain in energy by hydrogen bonding (λ^*n*H_2_O^) is to be evaluated
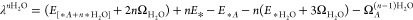
7with λ^0H_2_O^ = Ω_*A*_^0H_2_O^ = 0. We note that eq 7 considers that interfacial water molecules always have three hydrogen bonds. If λ^*n*H_2_O^ ≤ 0, the system is stabilized by the additional water molecule, and the iterative process continues.

**Figure 4:**
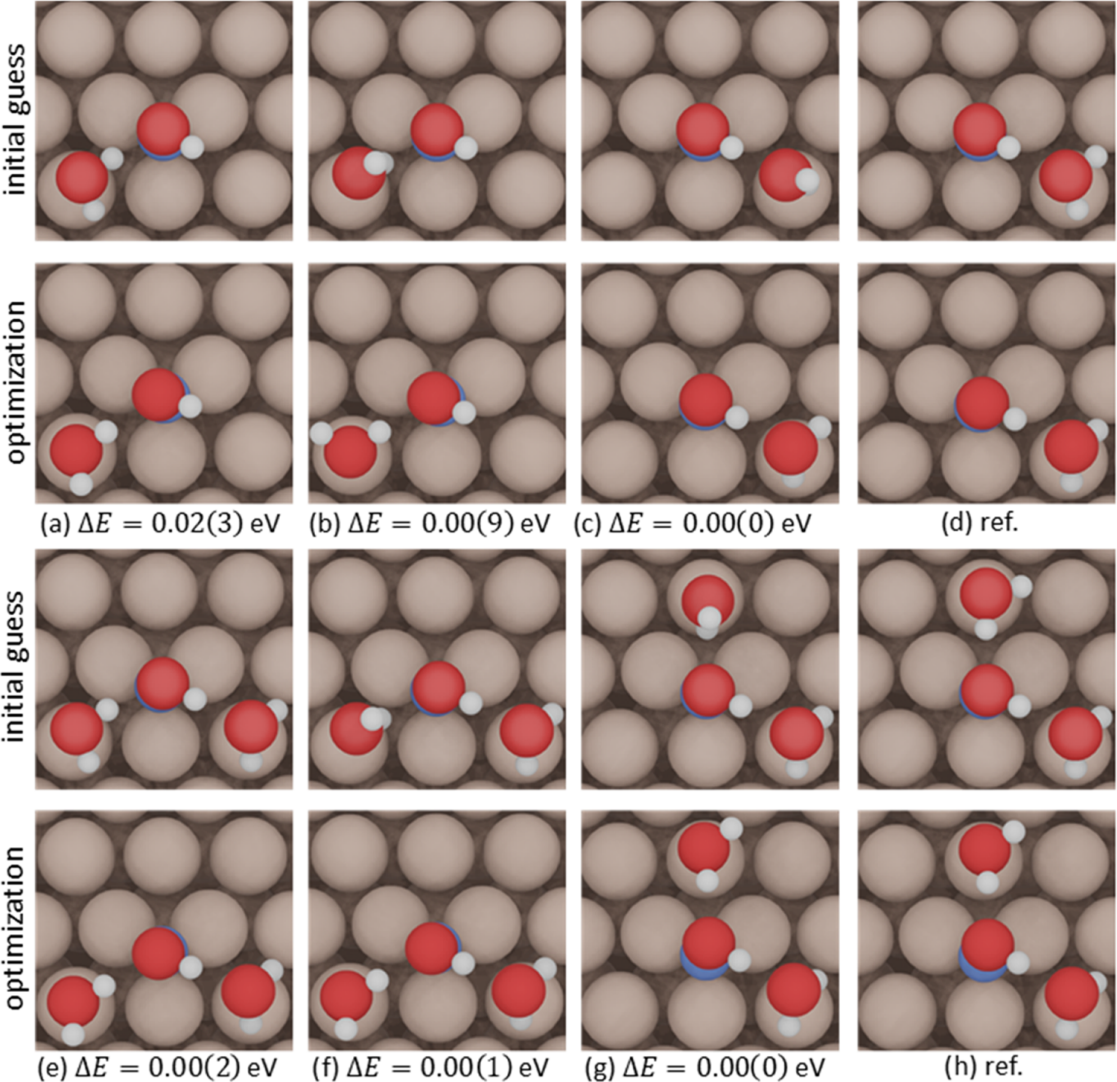
Initial and optimized geometry configurations when microsolvating *NOH on Cu(111) with one (a–d) and two (e–h) water molecules.

To illustrate the use of the method, we consider the solvation of the species *NOH on Cu(111), the adsorption energies of which with PBE and PBE-D3 are reported in Table S6. The calculated Ω_H_2_O_ for this facet is −0.24 ± 0.04 eV (Table S2). We adsorbed the first water molecule on top sites in the vicinity of the adsorbate, starting from four possible initial guesses: two configurations with the water molecule as a hydrogen-bond donor and two as an acceptor. One of the configurations has the water molecule parallel to the surface plane and the other has it perpendicular, see Figure 4a–d. Upon relaxation, we took the most stable configuration (Figure 4c). For this water molecule and *NOH, eq 6 gives a solvation energy of Ω_NOH_^1H_2_O^ = −0.29 eV and eq 7 gives a gain of λ^1H_2_O^ = −0.05 eV ≤ 0. Hence, the first water molecule effectively solvates *NOH, and we can add a second one, again considering different initial guesses for its location and orientation (Figure 4e–h). For the second addition on the most stable configuration, we found Ω_NOH_^2H_2_O^ = −0.30 eV and λ^2H_2_O^ = 0.17 eV ≥ 0 such that the second water molecule does not belong to the first solvation shell of *NOH on Cu(111), and the iterative process ends.

For completeness, we tested the above procedure with PBE-D3 with zero damping and BJ damping. With zero damping, we obtained Ω_H_2_O,D3_ = −0.23 ± 0.05 eV (Table S2), Ω_NOH,D3_^1*H*_2_*O*^ = −0.29 eV, and λ_D3_^1H_2_O^ = −0.06 eV, so the first water molecule is again part of the innermost solvation shell. For the second water molecule, we obtained Ω_NOH,D3_^2H_2_O^ = −0.33 eV and λ_D3_^2H_2_O^ = 0.13 eV ≥ 0 so that the second water molecule is not part of the innermost solvation shell.

For PBE-D3 with BJ damping, Ω_H_2_O,D3-BJ_ = −0.23 ± 0.04 eV (Table S2), Ω_NOH-D3-BJ_^1H_2_O^ = −0.28 eV, and λ_D3-BJ_^1H_2_O^ = −0.04 eV such that the first water molecule passes the test. For the second one, Ω_NOH,D3-BJ_^2H_2_O^ = −0.34 eV and λ_D3-BJ_^2H_2_O^ = 0.13 eV ≥ 0. Hence, the solvation shell contains only one water molecule. In summary, *NOH on Cu(111) is directly solvated by a single water molecule according to both PBE and PBE-D3.

It is noteworthy that the PBE and PBE-D3 water self-solvation energies and solvation energies are practically the same (Table S4). Thus, the method weighs well hydrogen bonds between the adsorbate and the solvating environment with or without dispersion corrections. However, a word of warning is necessary here: when solvation energies are close to the water self-solvation energy (Ω_*A*_^*n*H_2_O^ ≈ Ω_H_2_O_), it may happen that *n* changes when incorporating dispersion contributions. This is because of the small variations in the results between PBE and PBE-D3, as exemplified in Table S4 for *NHO on Pt(111).

Recent studies for Pt and Cu estimated the influence of water molecules located in the second solvation shell on the solvation energies. Comparing the results of a full water bilayer and microsolvation,^11,22^ differences on the scale of 0.05 eV were observed. This suggests that hydrogen bonding has a minor effect beyond the first solvation shell such that the water molecules around the adsorbate do not need to be solvated for the model to produce converged adsorbate solvation energies. For this reason, we have not included the impact of the second solvation shell here. For PBE, we tested the contribution of the second solvation shell by sequentially adding two water molecules interacting with the water molecule that solvates *NOH on Cu(111). As reported in Table S5, the solvation energy of *NOH assessed with a single water molecule in the first solvation shell is −0.29 eV. The solvation energy of *NOH after adding a water molecule in the second solvation shell is −0.27 eV, and it is −0.24 eV when another water molecule is added to the second solvation shell; see Figure S6 for details on the relaxed atomic configurations. Hence, differences in the range of 0.05 eV can be expected, as observed in previous microsolvation works.^11,22^

## Conclusions

To summarize and conclude, we dissected an inexpensive, iterative microsolvation method for the description of solvent–adsorbate interactions. At the core of the method there are two salient factors. The first one is the water self-solvation energy, which can be assessed using clusters of only four water molecules adsorbed on transition-metal surfaces. The second one is adsorbate–water interactions, which are evaluated by determining the number of water molecules located in the first solvation shell of the adsorbate that actively contribute to its stabilization through hydrogen bonding. Although including dispersion is generally regarded as a must for modeling bulk water, we verified that it is generally unnecessary to evaluate the water self-solvation energies and adsorbate solvation energies. Nevertheless, caution should be exercised when the water–water and water–adsorbate interactions are numerically similar. Our findings help save some computational time and, more importantly, avoid the use of dispersion corrections when strongly chemisorbed species coadsorb with water. In such systems, a fair assessment of adsorption energies that enables a predictive design of (electro)catalysts may be impeded by using overly stabilizing dispersion corrections.

## Supporting Information Available

Full computational details; tabulated water self-solvation energies; solvation energies, and water adsorption energies (PDF)
