# Supplementary material for: Evaluating Adsorbate–Solvent Interactions: Are Dispersion Corrections Necessary?
Source: J Phys Chem C Nanomater Interfaces. 2023 May 19;127(21):10134–9. doi: 10.1021/acs.jpcc.3c02934 (PMC10241112; doi:10.1021/acs.jpcc.3c02934)
Supplement: Supplementary file 1 — jp3c02934_si_001.pdf [file jp3c02934_si_001.pdf]

Supporting Information

Evaluating Adsorbate-Solvent Interactions: Are Dispersion Corrections Necessary?

Eleonora Romeo,[a] Francesc Illas,[a,*] and Federico Calle-Vallejo[b,c,*]

[a]Departament de Ciència de Materials i Química Física & Institut de Química Teòrica i Computacional (IQTCUB), Universitat de Barcelona, C/ Martí i Franquès 1, 08028 Barcelona, Spain.

[b]Nano-Bio Spectroscopy Group and European Theoretical Spectroscopy Facility (ETSF), Department of Polymers and Advanced Materials: Physics, Chemistry and Technology, University of the Basque Country UPV/EHU, Av. Tolosa 72, 20018 San Sebastián, Spain.

[c]IKERBASQUE, Basque Foundation for Science, Plaza de Euskadi 5, 48009 Bilbao, Spain.

***Corresponding authors:** francesc.illas@ub.edu ; federico.calle@ehu.es

Index of Contents

S1. Computational details 2

S2. Tabulated water self-solvation energies 5

S3. Solvation energies 9

S4. Adsorption energies 11

S5. References 13

S1. Computational details

All energy values and minimum energy structures were obtained from DFT calculations carried out with the VASP code.[1] The Perdew-Burke-Ernzerhof (PBE) exchange-correlation functional[2] was chosen to compute the total energy of the scrutinized systems, as it was found to accurately describe the three series of transition metals[3,4] and their low Miller index surfaces.[5] A plane-wave basis set with a kinetic energy cutoff of 450 eV was used to expand the valence electron density. The effect of the atomic cores on the valence electron density was accounted for through the projector augmented-wave (PAW) method[6] as implemented in VASP.[7] The Methfessel-Paxton approach was used to ease the convergence of the self-consistent field, a smear of the Fermi level with $k_BT = 0.2$ eV was used and, upon convergence, the total energies were extrapolated to 0 K. For Co and Ni, all the calculations were spin unrestricted.

Periodic slabs of four atomic layers were used to represent the extended surfaces, which were modelled from the bulk with the converged PBE lattice constants reported in Table S1. For the (111) and (100) surfaces, (4×4) supercells were used that contained 16 atoms per atomic layer. A representation of the slabs is provided in Figure S1. Co was assumed to be an fcc metal and, based on previous works,[8] we do not expect significant changes as a result of that approximation. The numerical integration in the reciprocal space was carried out using Monkhorst-Pack[9] grids of 3×3×1 for the (100) and (111) facets, which guaranteed convergence of the adsorption energies within ±0.05 eV. To avoid spurious electrostatic interactions, a vacuum of more than 15 Å has been applied between periodically repeated images in the vertical direction, and a dipole correction was applied, as well. For the geometry optimizations, the conjugate gradient optimization algorithm was used, with iterations performed until the maximal residual force on all atoms was below 0.05 eV $Å^{-1}$.

Boxes of 9 Å × 10 Å ×11 Å were used to calculate gas-phase H_2O, considering only the Γ-point, using Gaussian smearing and $k_BT = 0.001$ eV with further extrapolation to 0 K. On top of PBE, dispersion effects were included via the D3 correction method of Grimme (PBE-D3) both with zero damping and with Becke-Johnson (BJ) damping, as implemented in VASP.[10–12]

We calculated the water self-solvation criteria using a cluster of four water molecules that are both the solvent (peripheral molecules) and the adsorbate (central molecule), and are in contact with the meta surface. We compute three different configurations for the central water molecule: one with both hydrogen atoms in a plane parallel to the surface (denoted “parallel”), one with a hydrogen atom oriented upwards, and one a hydrogen atom oriented downwards,[13]

as represented in Figure S2. With these three different results, we can compute the error bar for each water self-solvation criterion.

Table S1. Lattice constant of the metals under study as predicted by PBE.

Metal	Lattice constant (Å)
Co	3.52
Ni	3.52
Cu	3.64
Rh	3.84
Pd	3.96
Ag	4.17
Ir	3.88
Pt	3.98
Au	4.18

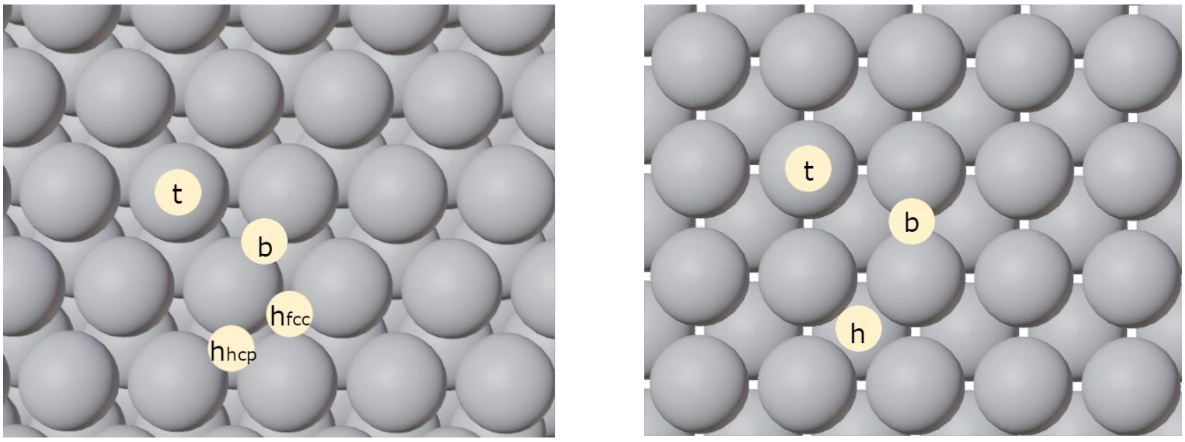

Figure S1. Top view of (111) (left) and (100) (right) facets under study. The monodentate adsorption sites are indicated and marked as follows: fcc threefold hollow site (h_{fcc}), hcp threefold hollow site (h_{hcp}), fourfold hollow sites (h), atop site (top), bridge site (b).

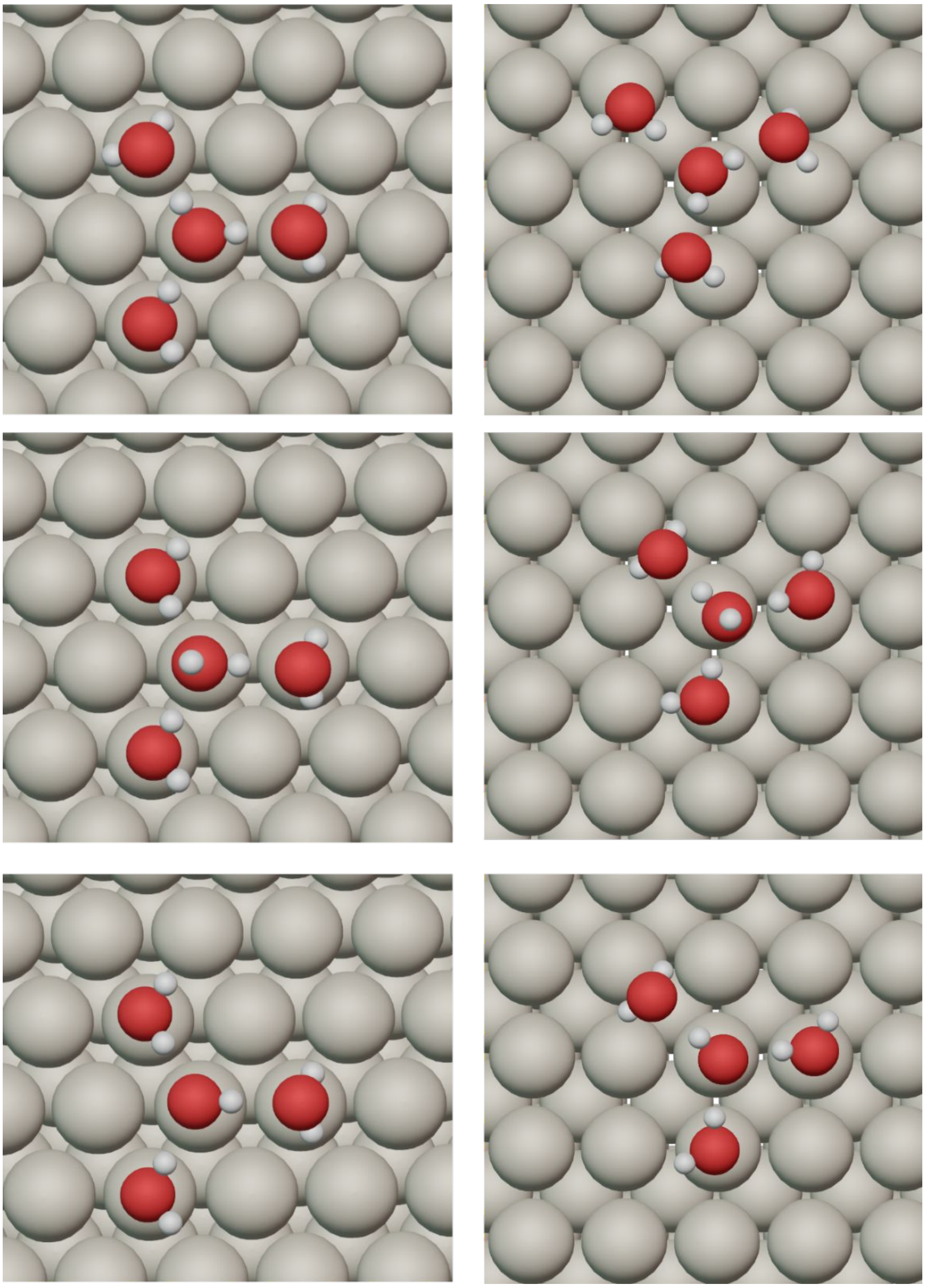

Figure S2. Clusters of four water molecules with the central molecule in a configuration with the hydrogen atoms parallel to the surface (top panels), with one hydrogen atom pointing upwards (middle panels), one hydrogen atom pointing downwards (bottom panels). The schematics for the (111)/(100) facets are on the left/right.

S2. Tabulated water self-solvation energies

Table S2. Water self-solvation energy (in eV) for the (111) facet of nine transition metals without dispersion contributions, with D3 dispersion contributions with zero damping and BJ damping. Parallel and Up/down is as defined above.

metal	$\Omega_{H_2O,parallel}$	$\Omega_{H_2O,\ up}$	$\Omega_{H_2O,down}$	Ω_{H_2O}	±
No dispersion					
Co	-0.24	-0.19	-0.15	-0.19	0.05
Ni	-0.23	-0.18	-0.15	-0.19	0.04
Cu	-0.28	-0.24	-0.20	-0.24	0.04
Rh	-0.20	-0.14	-0.11	-0.15	0.05
Pd	-0.23	-0.19	-0.16	-0.20	0.03
Ag	-0.26	-0.23	-0.20	-0.23	0.03
Ir	-0.21	-0.15	-0.11	-0.16	0.05
Pt	-0.26	-0.21	-0.19	-0.22	0.04
Au	-0.26	-0.23	-0.21	-0.24	0.03
PBE-D3 with zero damping					
Co	-0.25	-0.17	-0.14	-0.19	0.06
Ni	-0.25	-0.17	-0.15	-0.19	0.05
Cu	-0.29	-0.22	-0.19	-0.23	0.05
Rh	-0.20	-0.13	-0.08	-0.14	0.06
Pd	-0.25	-0.19	-0.17	-0.20	0.04
Ag	-0.27	-0.23	-0.20	-0.23	0.04
Ir	-0.21	-0.13	-0.10	-0.15	0.06
Pt	-0.27	-0.20	-0.19	-0.22	0.04
Au	-0.28	-0.23	-0.22	-0.24	0.03
PBE-D3 with BJ damping					
Co	-0.25	-0.17	-0.13	-0.18	0.06
Ni	-0.24	-0.17	-0.14	-0.18	0.05
Cu	-0.28	-0.22	-0.19	-0.23	0.04
Rh	-0.20	-0.12	-0.07	-0.13	0.06
Pd	-0.24	-0.17	-0.16	-0.19	0.04
Ag	-0.27	-0.22	-0.20	-0.23	0.04
Ir	-0.20	-0.12	-0.09	-0.14	0.06
Pt	-0.26	-0.19	-0.18	-0.21	0.04
Au	-0.28	-0.21	-0.21	-0.23	0.03

Table S3. Water self-solvation energy (in eV) for the (100) facet without dispersion contributions, with D3 dispersion contributions with zero damping and BJ damping.

metal	$\Omega_{H_2O, parallel}$	$\Omega_{H_2O, up}$	$\Omega_{H_2O, down}$	Ω_{H_2O}	±
No dispersion					
Co	-0.16	-0.13	-0.10	-0.13	0.03
Ni	-0.13	-0.14	-0.10	-0.12	0.02
Cu	-0.21	-0.21	-0.16	-0.19	0.03
Rh	-0.13	-0.16	-0.12	-0.13	0.02
Pd	-0.19	-0.22	-0.17	-0.19	0.02
Ag	-0.24	-0.24	-0.19	-0.22	0.02
Ir	-0.12	-0.13	-0.09	-0.11	0.02
Pt	-0.24	-0.25	-0.20	-0.23	0.02
Au	-0.23	-0.24	-0.20	-0.22	0.02
PBE-D3 with zero damping					
Co	-0.18	-0.13	-0.10	-0.14	0.04
Ni	-0.16	-0.12	-0.11	-0.13	0.03
Cu	-0.23	-0.20	-0.17	-0.20	0.03
Rh	-0.16	-0.16	-0.12	-0.15	0.02
Pd	-0.20	-0.23	-0.19	-0.21	0.02
Ag	-0.26	-0.25	-0.21	-0.24	0.03
Ir	-0.09	-0.12	-0.12	-0.11	0.01
Pt	-0.25	-0.27	-0.23	-0.25	0.02
Au	-0.24	-0.26	-0.22	-0.24	0.02
PBE-D3 with BJ damping					
Co	-0.17	-0.13	-0.10	-0.13	0.04
Ni	-0.16	-0.13	-0.10	-0.13	0.03
Cu	-0.22	-0.20	-0.17	-0.20	0.03
Rh	-0.14	-0.15	-0.10	-0.13	0.02
Pd	-0.24	-0.22	-0.18	-0.21	0.03
Ag	-0.27	-0.25	-0.21	-0.24	0.03
Ir	-0.12	-0.11	-0.07	-0.10	0.02
Pt	-0.23	-0.26	-0.22	-0.23	0.02
Au	-0.24	-0.27	-0.22	-0.24	0.02

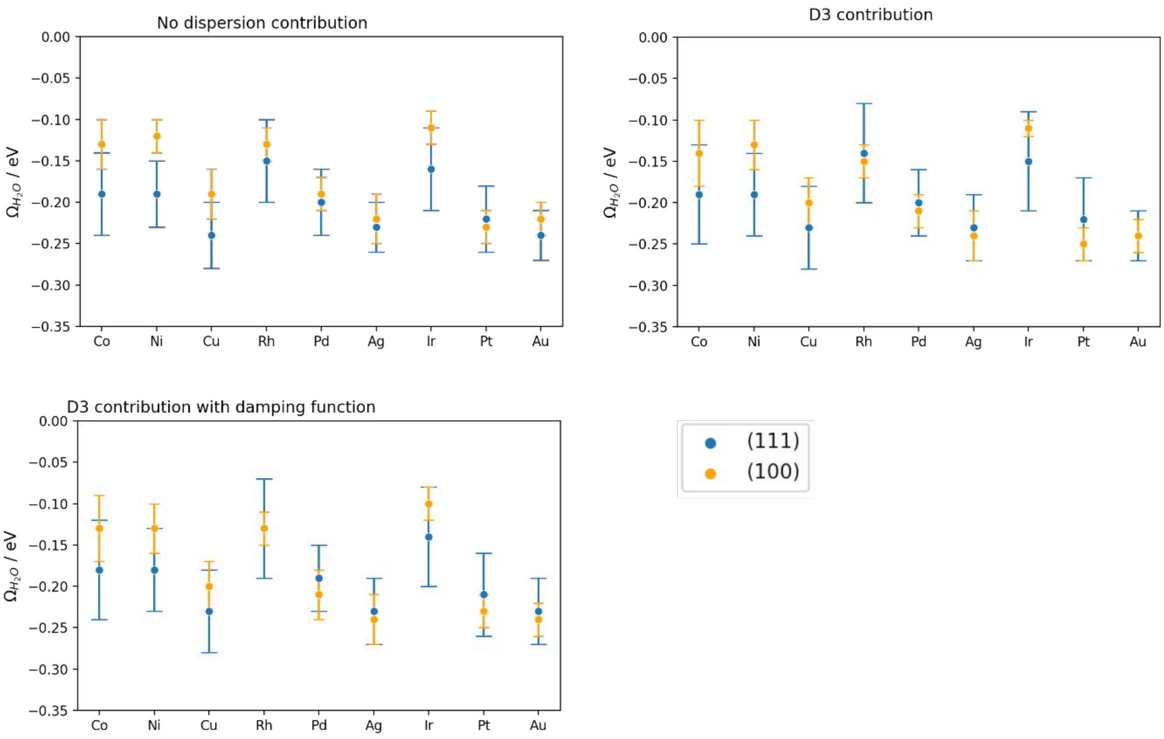

Figure S3. Water self-solvation energy calculated without dispersion contributions (upper-left panel), with D3 dispersion corrections with zero damping (upper-right panel) and D3 with BJ damping (bottom panel).

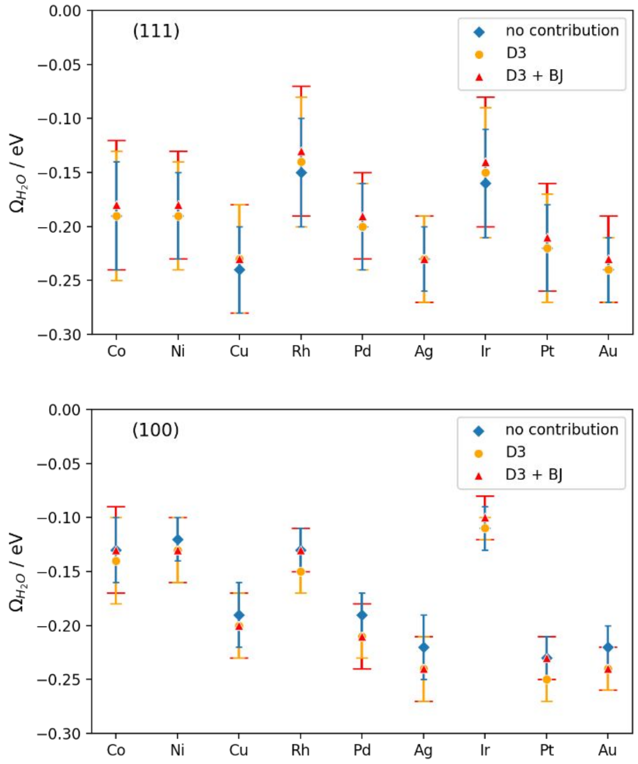

Figure S4. Comparison of water self-solvation energies for (111) and (100) facets calculated without dispersion corrections (in blue), with D3 dispersion corrections with zero damping (in yellow) and D3 dispersion corrections with BJ damping (in red).

S3. Solvation energies

Table S4. Examples of computed solvation energies (all values are in eV).

method	Ω^{1H_2O}	λ^{1H_2O}	Ω^{2H_2O}	λ^{2H_2O}	$\Omega_A^{nH_2O}$
		*NO on Cu(111)			
PBE	-0.21	0.03	-0.42	0.27	-0.21
PBE-D3	-0.22	0.01	-0.46	0.23	-0.22
PBE-D3(BJ)	-0.24	0.00	-0.48	0.22	-0.24
		*NOH on Cu(111)			
PBE	-0.29	-0.05	-0.30	0.17	-0.29
PBE-D3	-0.29	-0.06	-0.33	0.13	-0.29
PBE-D3(BJ)	-0.28	-0.04	-0.34	0.13	-0.28
		*NHO on Pt(111)			
PBE	-0.19	0.03	-0.13	0.31	-0.19
PBE-D3	-0.17	0.05	-	-	0.00
PBE-D3(BJ)	-0.16	0.05	-	-	0.00

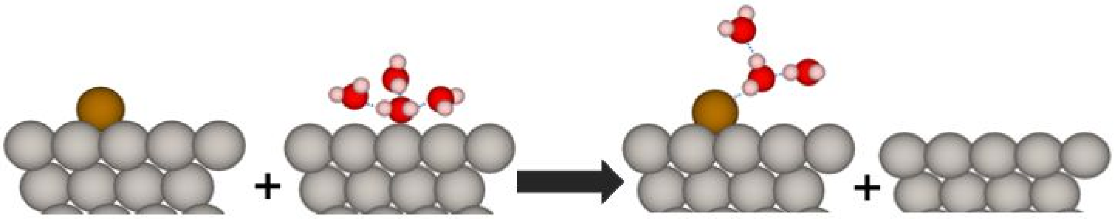

Figure S5. Representation of the solvation process in Equation 5 of the main text for a generic adsorbate (in ochre) by one water molecule. For the water molecules, it has to be considered an equal number of hydrogen bonds in the reagents and products. Hence, in the evaluation of λ^{nH_2O} in the main text, $3n\Omega_{H_2O}$ indicates that each molecule has three hydrogen bonds in the reactants. Conversely, in the products $2n\Omega_{H_2O}$ indicates that each water molecule coordinated with the adsorbate makes two hydrogen bonds with water and one with the adsorbate.

Table S5. Solvation energies (in eV) computed with PBE calculated with a single water molecule in the first solvation shell and sequentially adding one and two water molecules in the second solvation shell.

	$\Omega_A^{1H_2O}$	$\Omega_A^{1H_2O + 1H_2O}$	$\Omega_A^{1H_2O + 2H_2O}$
*NOH @ Cu(111)	-0.29	-0.27	-0.24

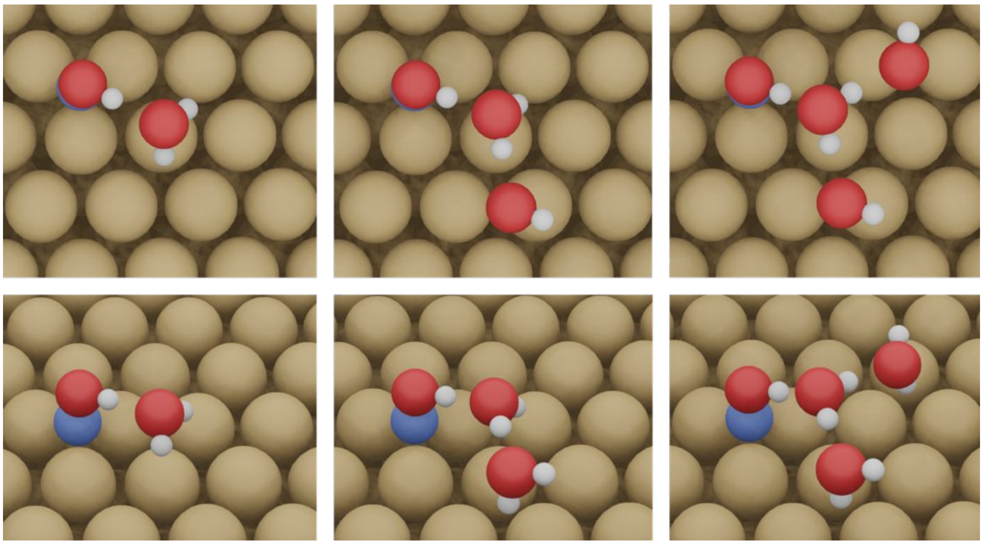

Figure S6. Top and side representations of the solvating environment for *NOH on Cu(111) calculated with PBE with one water molecule in the first solvation shell (left); one water molecule in the first solvation shell and another in the second solvation shell (center); one water molecule in the first solvation shell and two water molecules in the second solvation shell (right).

S4. Adsorption energies

Table S6. Adsorption energies for *NO@Cu(111), *NOH@Cu(111) and *NHO@Pt(111) (i) without dispersion contributions; (ii) with D3 dispersion corrections with zero damping; and (iii) with D3 dispersion contributions with BJ damping.

**adsorbate**	ΔE_{ads} (i)	ΔE_{ads} (ii)	ΔE_{ads} (iii)	(ii) – (i)	(iii) – (i)
*NO@Cu(111)	-0.56	-0.81	-0.86	-0.25	-0.30
*NOH@Cu(111)	-0.53	-0.84	-0.88	-0.31	-0.35
*NHO@Pt(111)	-1.24	-1.57	-1.62	-0.33	-0.38

Table S7. Water adsorption energies (in eV) for the (111) facets (i) without dispersion contributions; (ii) with D3 dispersion corrections with zero damping; and (iii) with D3 dispersion contributions with BJ damping.

**metal**	ΔE_{ads} (i)	ΔE_{ads} (ii)	ΔE_{ads} (iii)	(ii) – (i)	(iii) – (i)
Co	-0.27	-0.49	-0.48	-0.22	-0.21
Ni	-0.29	-0.52	-0.51	-0.23	-0.22
Cu	-0.17	-0.42	-0.40	-0.25	-0.22
Rh	-0.34	-0.55	-0.58	-0.21	-0.24
Pd	-0.26	-0.47	-0.49	-0.21	-0.23
Ag	-0.13	-0.31	-0.31	-0.18	-0.18
Ir	-0.31	-0.55	-0.57	-0.24	-0.25
Pt	-0.22	-0.46	-0.46	-0.24	-0.24
Au	-0.11	-0.31	-0.31	-0.20	-0.19
			average	-0.22 ± 0.02	-0.22 ± 0.02

Table S8. Water adsorption energies (in eV) for the (100) facets (i) without dispersion contributions; (ii) with D3 dispersion corrections with zero damping; (iii) with D3 dispersion corrections with BJ damping.

**metal**	ΔE_{ads} (i)	ΔE_{ads} (ii)	ΔE_{ads} (iii)	(ii) – (i)	(iii) – (i)
Co	-0.36	-0.56	-0.55	-0.20	-0.19
Ni	-0.38	-0.59	-0.58	-0.22	-0.20
Cu	-0.24	-0.46	-0.43	-0.22	-0.20
Rh	-0.36	-0.55	-0.58	-0.19	-0.22
Pd	-0.27	-0.45	-0.46	-0.18	-0.20
Ag	-0.15	-0.30	-0.31	-0.15	-0.16
Ir	-0.43	-0.65	-0.67	-0.22	-0.24
Pt	-0.26	-0.46	-0.47	-0.20	-0.21
Au	-0.14	-0.32	-0.31	-0.17	-0.17
			average	-0.20 ± 0.02	-0.20 ± 0.02

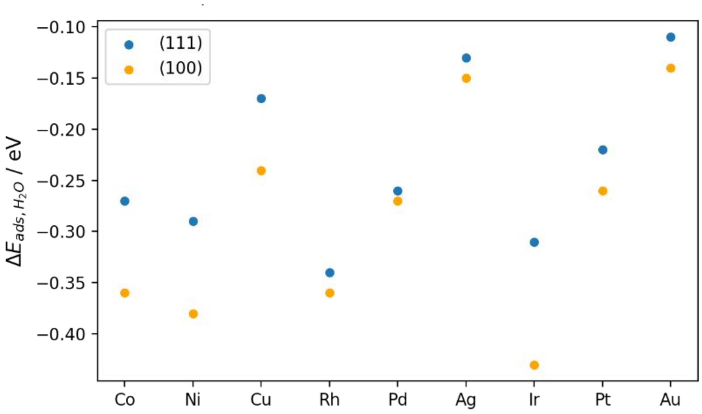

Figure S7. Adsorption energies of water on (111) and (100) surfaces of transition metals without dispersion contributions. In all cases, the (100) facet adsorbs water more strongly than the (111) facet.

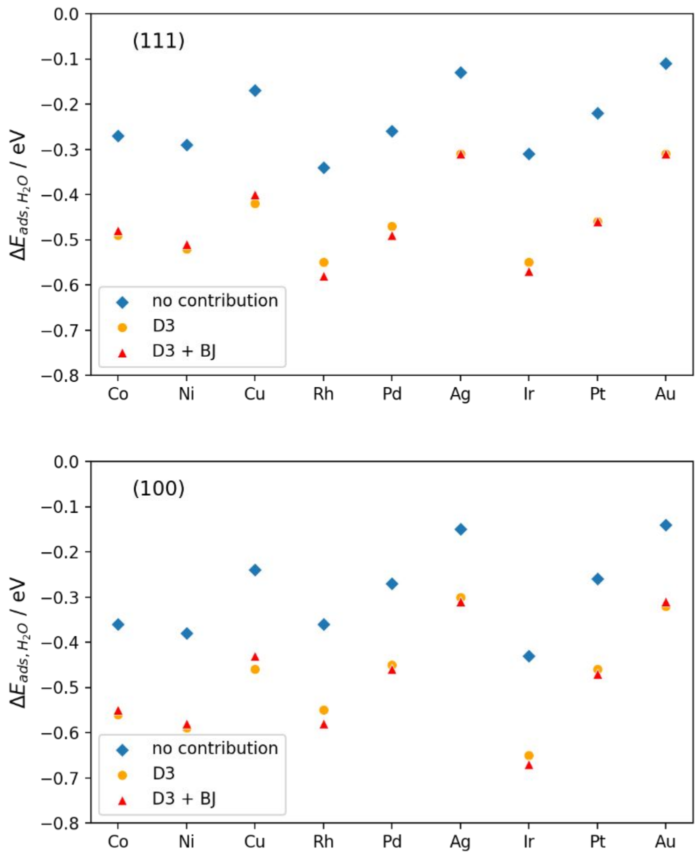

Figure S8. Comparison of water adsorption energy for the (111) and (100) facets of transition metals calculated without dispersion contribution (in blue), with D3 dispersion corrections with zero damping (in yellow), and with D3 dispersion corrections with BJ damping (in red).

S5. References

(1) Kresse, G.; Furthmüller, J. Efficient Iterative Schemes for Ab Initio Total-Energy Calculations Using a Plane-Wave Basis Set. Phys. Rev. B 1996, 54 (16), 11169–11186.

(2) Perdew, J. P.; Burke, K.; Ernzerhof, M. Generalized Gradient Approximation Made Simple. Phys. Rev. Lett. 1996, 77 (18), 3865–3868.

(3) Janthon, P.; Kozlov, S. M.; Viñes, F.; Limtrakul, J.; Illas, F. Establishing the Accuracy of Broadly Used Density Functionals in Describing Bulk Properties of Transition Metals. J. Chem. Theory Comput. 2013, 9 (3), 1631–1640.

(4) Janthon, P.; Luo, S. (Andy); Kozlov, S. M.; Viñes, F.; Limtrakul, J.; Truhlar, D. G.; Illas, F. Bulk Properties of Transition Metals: A Challenge for the Design of Universal Density Functionals. J. Chem. Theory Comput. 2014, 10 (9), 3832–3839.

(5) Vega, L.; Ruvireta, J.; Viñes, F.; Illas, F. Jacob's Ladder as Sketched by Escher: Assessing the Performance of Broadly Used Density Functionals on Transition Metal Surface Properties. J. Chem. Theory Comput. 2018, 14 (1), 395–403.

(6) Blöchl, P. E. Projector Augmented-Wave Method. Phys. Rev. B 1994, 50 (24), 17953–17979.

(7) Kresse, G.; Joubert, D. From Ultrasoft Pseudopotentials to the Projector Augmented-Wave Method. Phys. Rev. B 1999, 59 (3), 1758–1775.

(8) Calle-Vallejo, F.; Koper, M. T. M. Accounting for Bifurcating Pathways in the Screening for CO2 Reduction Catalysts. ACS Catal. 2017, 7 (10), 7346–7351.

(9) Monkhorst, H. J.; Pack, J. D. Special Points for Brillouin-Zone Integrations. Phys. Rev. B 1976, 13 (12), 5188–5192.

(10) Grimme, S. Accurate description of van der Waals complexes by density functional theory including empirical corrections. J. Comput. Chem. 2004, 25 (12), 1463–1473.

(11) Grimme, S.; Antony, J.; Ehrlich, S.; Krieg, H. A Consistent and Accurate Ab Initio Parametrization of Density Functional Dispersion Correction (DFT-D) for the 94 Elements H-Pu. J. Chem. Phys. 2010, 132 (15), 154104.

(12) Grimme, S.; Ehrlich, S.; Goerigk, L. Effect of the Damping Function in Dispersion Corrected Density Functional Theory. J. Comput. Chem. 2011, 32 (7), 1456–1465.

(13) Rendón-Calle, A.; Builes, S.; Calle-Vallejo, F. Substantial Improvement of Electrocatalytic Predictions by Systematic Assessment of Solvent Effects on Adsorption Energies. Appl. Catal. B Environ. 2020, 276, 119147.
